# Pharyngeal perforation following laryngoscopy in a patient with dysphagia secondary to diffuse idiopathic skeletal hyperostosis: A case report

**DOI:** 10.1097/MD.0000000000021526

**Published:** 2020-07-31

**Authors:** Hongxiang Gao, Xueju Li, Cunping Wang

**Affiliations:** Department of Spine, Zaozhuang Municipal Hospital, Zaozhuang, Shandong, China.

**Keywords:** deglutition disorders, diffuse idiopathic skeletal, hyperostosis, laryngoscopy complication, pharyngeal perforation

## Abstract

**Rationale:**

Dysphagia is a common presenting symptom in elderly people. Nevertheless, dysphagia resulting from diffuse idiopathic skeletal hyperostosis (DISH) of patients’ cervical spine may be due to several factors. Despite computed tomography scan showing the size and shape of osteophytes, endoscopy may be necessary to exclude other intrinsic causes of dysphagia. Due to the anatomic variation of the pharynx secondary to DISH, patients undergoing endoscopy are at risk of perforation. Once perforation occurs, inappropriate treatments may finally lead to an irretrievable outcome.

**Patient concerns:**

A 58-year-old male patient with a 20-day history of dysphagia initially visited an ear-nose-throat (ENT) doctor. He had no neck pain and no other history of cervical disease.

**Diagnosis:**

This patient with dysphagia due to DISH of the cervical spine underwent laryngoscopy to exclude other causes. Pharyngeal perforation resulted as a complication of the procedure.

**Interventions:**

The patient underwent laryngoscopy and biopsy by an ENT doctor to exclude intrinsic causes. After the procedure, a perforation was formed on the posterior wall of the pharynx. Conservative management, that is, 1 week of nothing per oral, and 1 month of antibiotics, was adopted. On the 30th day after the examination, the patient was voluntarily discharged from the hospital and recommended to take antibiotics orally.

**Outcomes:**

On the 56th day, the patient experienced fever and neck pain. Magnetic resonance imaging showed that the cervical vertebral bodies and spinal cord were infected. On the midday of the 60th day, the patient had a failed resuscitation and died.

**Lessons:**

DISH involving the cervical spine is a complicated cause of dysphagia. Due to the anatomic variation of the pharynx secondary to DISH, patients undergoing endoscopy are at risk of perforation. If other intrinsic causes of dysphagia have to be excluded with the aid of endoscopy, plain films and computed tomography images should be read carefully first. To minimize the risk of perforation, it is necessary to perform endoscopy extremely carefully, especially biopsy. Once perforation occurs, operative treatment may be more appropriate and effective.

## 1 Introduction

Diffuse idiopathic skeletal hyperostosis (DISH) was first described by Forestier and Rotes-Querol in 1950.^[1]^ It is characterized by excessive ligamentous calcification and ossification at the spinal and extra-spinal locations and prevalent within the elderly population. The most common clinical presentations of DISH are spinal stiffness and decreased range of motion secondary to the new bone and osteophyte formation. Occasionally, excessive skeletal hyperostosis of the cervical spine may cause symptoms of dysphagia. Most patients with dysphagia initially visit their ENT or gastroenterology doctors. Endoscopy is an important examination for diagnosis, but patients undergoing this procedure are at risk of perforation.^[2]^

The author shows a case of a 58-year-old male patient with dysphagia who developed perforation after laryngoscopic examination. Despite multiple conservative treatments for perforation, the cervical vertebral bodies were infected, and the infection even extended to the spinal cord. Consequently, the patient died. This case serves not only as an educational tool by bringing attention to an unintended consequence of a common complication of laryngoscopy in a common disorder, but it also provides significant information regarding the prevention and appropriate management of this complication.

## 2 Case presentation

**Figure 1:**
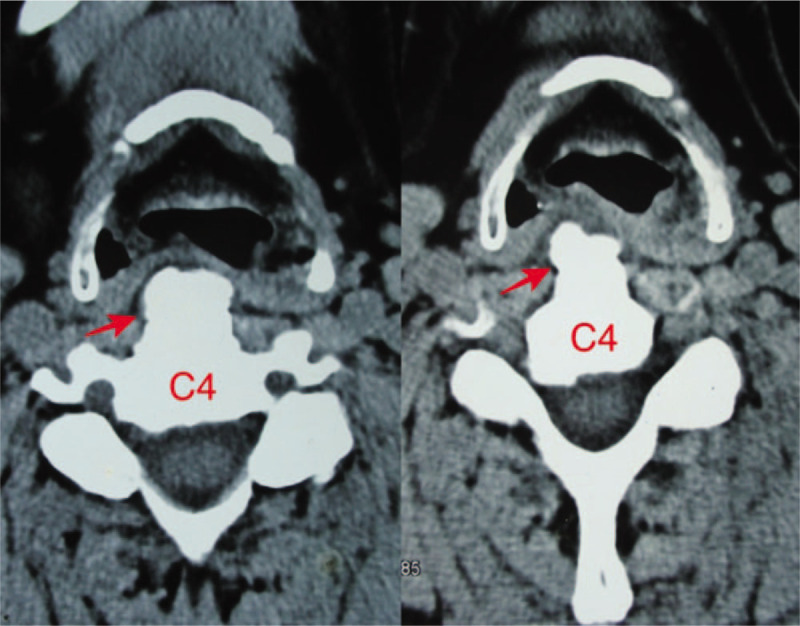
Axial CT slices through C4 in soft windows demonstrated the posterior wall of the pharynx was pushed to the pharyngeal cavity by large osteophytes (arrow). CT = computed tomography.

**Figure 2:**
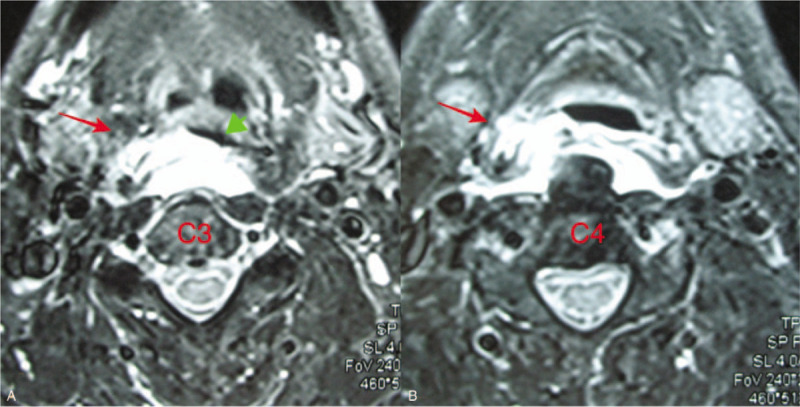
Axial MRI slices through C3 (A) and C4 (B) on T2-weighted images showed increased signal (arrow) between the posterior wall of the laryngopharynx and the cervical vertebral bodies. The pharyngeal cavity almost closed (arrowhead). MRI = magnetic resonance imaging.

A 58-year-old male patient presented with a 20-day history of dysphagia who initially visited an ENT doctor. A computed tomography (CT) scan of the pharynx showed a soft tissue protruding to the pharyngeal cavity associated with bulk osteophytes behind the posterior wall of the pharynx (Fig. 1). Nine days later, laryngoscopic examination was performed to determine the cause of the patient's symptoms. The examination showed a 1.5-cm-diameter mucosal tissue protruding from the pharynx, which was glossy and hyperemic and had no excretion. There were no motility disorders or tumors in the pharynx. At the same time, biopsy was performed to exclude other causes. On the second day after the examination, the patient experienced severe symptoms of odynophagia upon drinking water or liquid food. He was unable to cough and swallow spontaneously. Magnetic resonance imaging (MRI) of the pharynx was ordered to determine the causes. There was an increased signal between the posterior wall of the laryngopharynx and cervical vertebral bodies (C3–C5) on T2-weighted images (Fig. 2). According to MRI, odynophagia resulted from the perforation of the posterior wall of the laryngopharynx after biopsy. Consequently, the patient was kept nothing per oral (NPO), and antibiotics were administered to prevent possible infection. On the third day, the pathological report showed that the biopsy specimen was the connective tissue of coating squamous epithelium, glands, striated muscle, and a small number of fat cells. This result also demonstrated that the biopsy penetrated the posterior wall of the pharynx.

**Figure 3:**
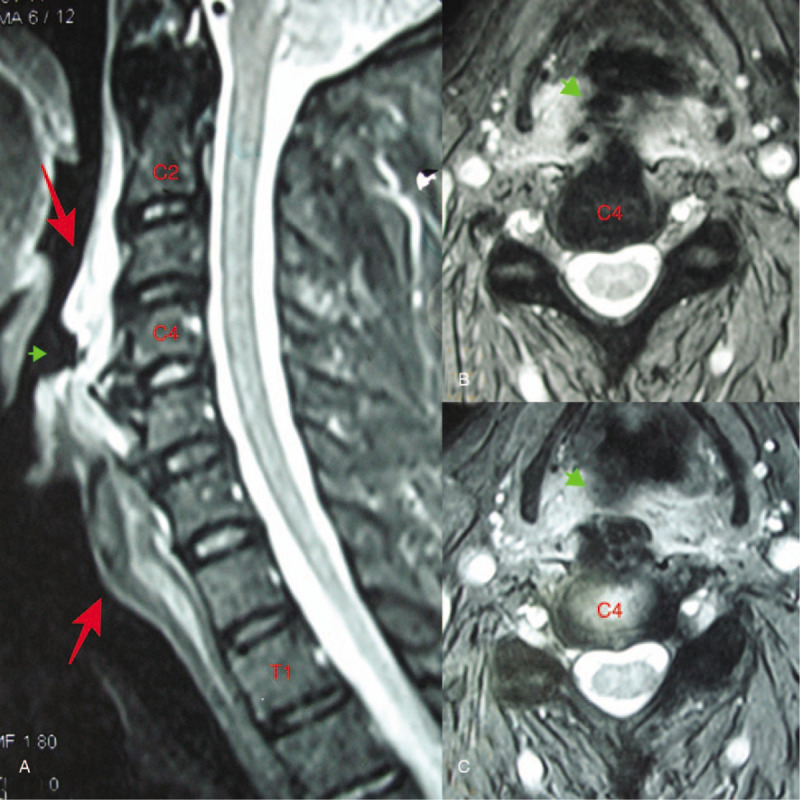
(A) Sagittal cervical spine MRI showed that an increased diffusion signal in the anterior soft tissue from the C2 to T1 cervical vertebral bodies on T2-weighted images and a small niche (arrowhead) on the posterior wall of the pharynx. (B and C) Axial MRI slices also showed a small niche (arrowhead) on the posterior wall of the pharynx. MRI = magnetic resonance imaging.

**Figure 4:**
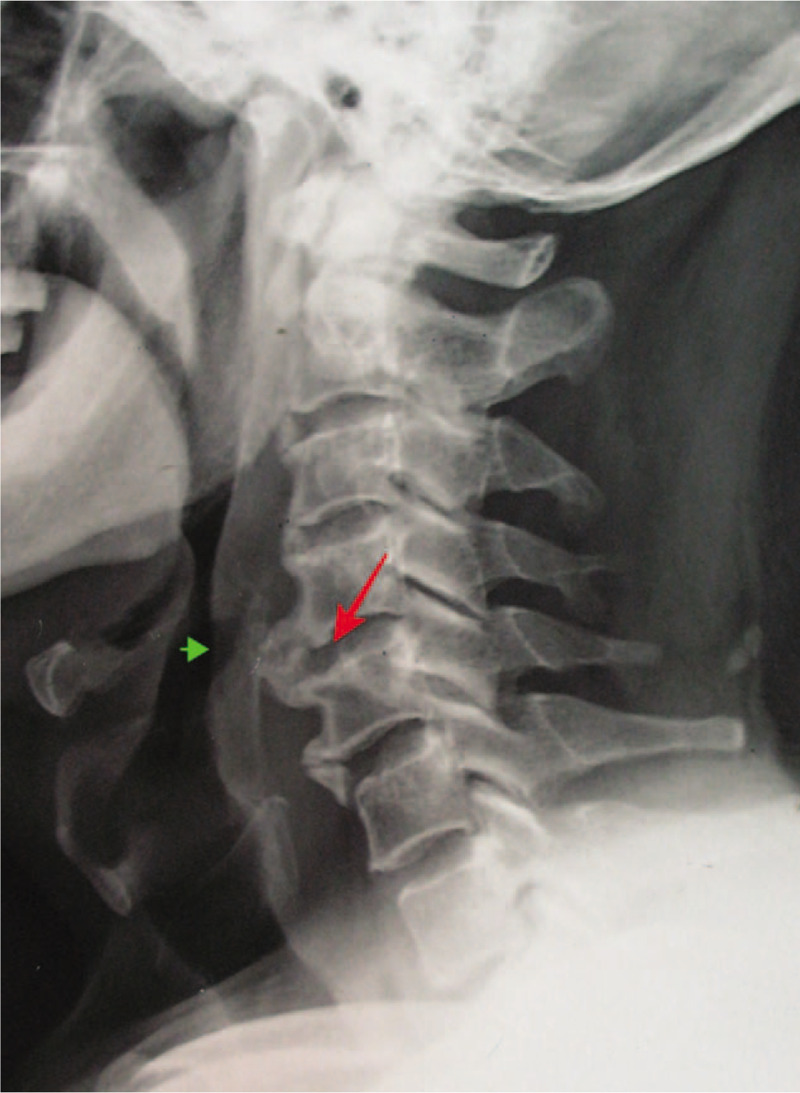
Lateral cervical spine radiograph showed a large flowing hyperostosis extended from the anterior aspect of C2 to C6, marked at the C4/C5 vertebrae (arrow). There was also a small niche shadow (arrowhead) on the posterior wall of the pharynx.

After 1 week of NPO and antibiotic administration, the patient was relieved of pain upon swallowing. He was then allowed to drink some water and had liquid diet. MRI of the cervical spine was ordered to reevaluate the anterior cervical vertebral bodies. Sagittal cervical spine MRI showed an increased diffusion signal in the anterior soft tissue from the C2 to T1 cervical vertebral bodies on T2-weighted images (Fig. 3). There was a minute niche on the posterior wall of the pharynx. A lateral cervical spine radiograph showed a large flowing hyperostosis extending from the anterior aspect of C2 to C6, predominantly at the C4/C5 vertebrae (Fig. 4). According to the outcome of these images, antibiotics were readministered.

On the 17th day, the patient visited another hospital and underwent laryngoscopy. The results showed that the biopsied mucosa developed into an ulcer. On the 20th day, his routine blood test showed a high leukocyte count (12.25 × 10^9^/L) with a high percentage of neutrophil granulocytes (81.74%). The erythrocyte sedimentation rate and high-sensitivity C-reactive protein level were 47 mm/h and 22.86 mg/L, respectively. Laboratory examinations implied that the patient still had infection. However, the doctor did not recommend other treatments aside from antibiotics. Soon afterward, the patient returned to the former hospital and continued to use antibiotics. On the 30th day, the patient was voluntarily discharged from the hospital and recommended to take antibiotics orally.

**Figure 5:**
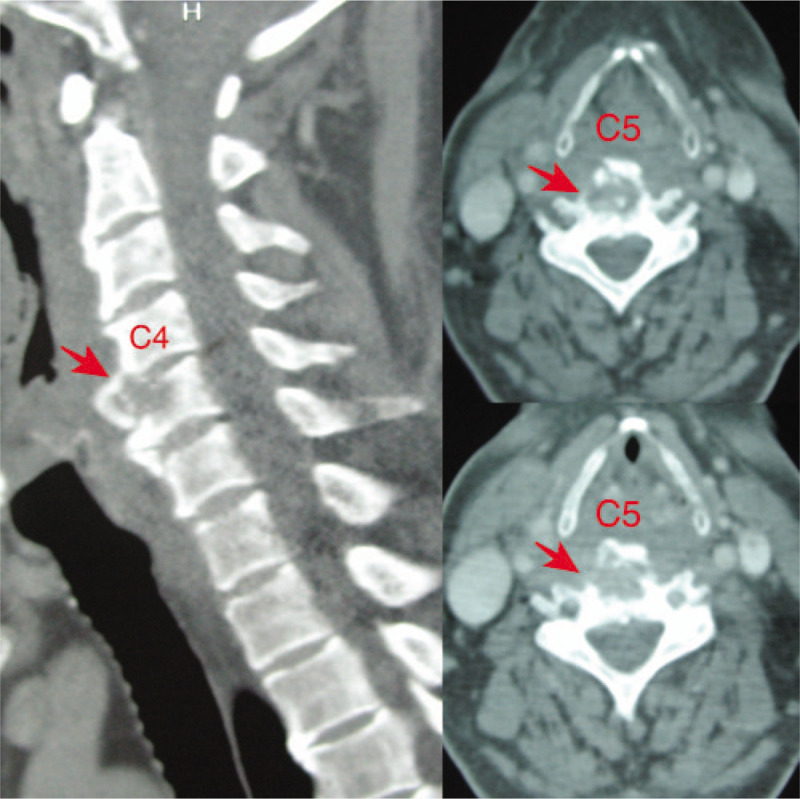
CT scan of the cervical spine showed that the C4 and C5 vertebral bodies were eroded and destroyed (arrow). CT = computed tomography.

**Figure 6:**
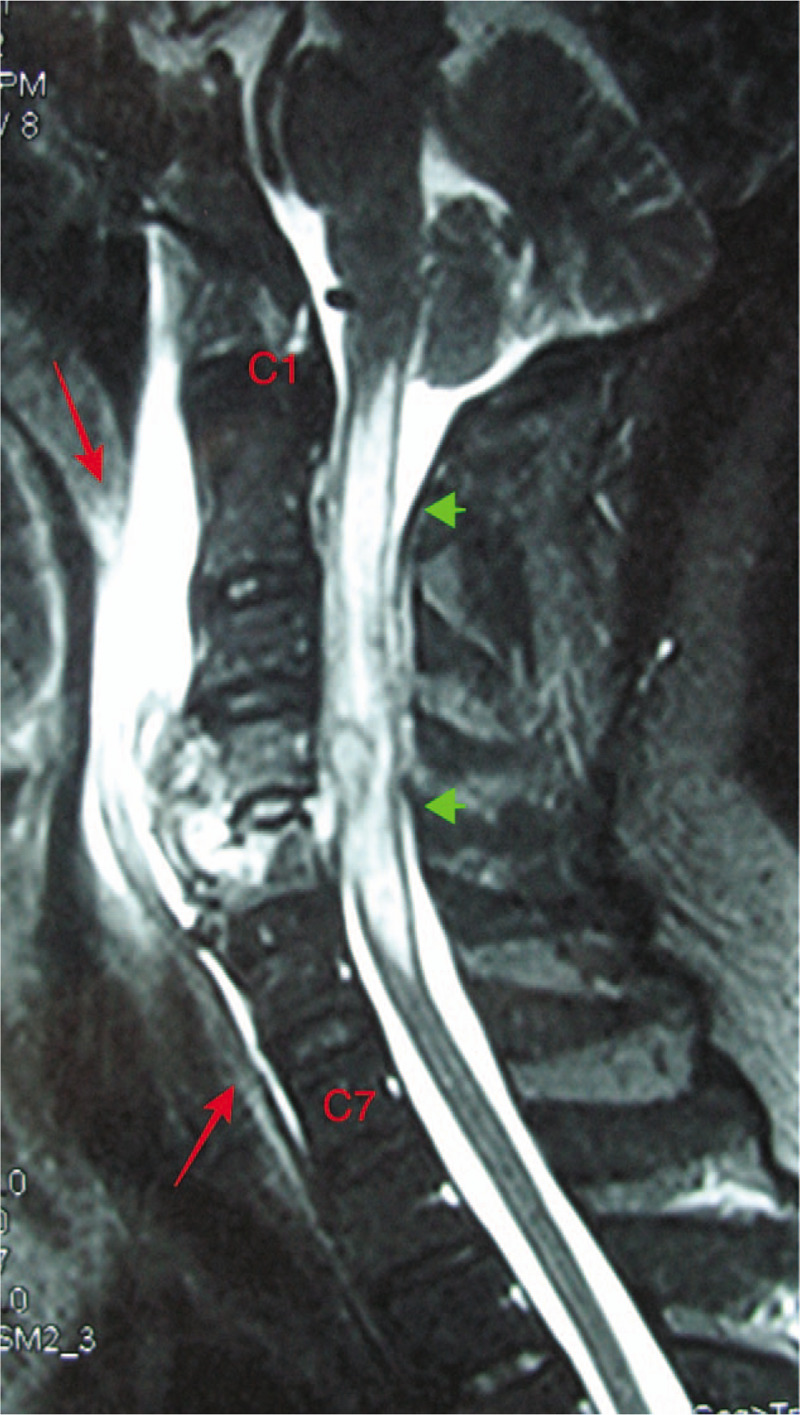
Sagittal cervical spine MRI on T2-weighted images showed that an increased diffusion signal in the C1–C6 spinal cord (arrowhead) and in the anterior soft tissue from the C1 to C7 cervical vertebral bodies (arrow). MRI = magnetic resonance imaging.

On the 56th day, the patient suddenly had fever and neck pain. He immediately returned to the second hospital and again underwent laryngoscopic examination. The results indicated that the ulcer almost healed. However, a CT scan of the cervical spine showed that the C4 and C5 vertebral bodies were eroded and destroyed (Fig. 5). On the 57th day, limb anesthesia occurred, and he could not move freely. On the 58th day, he visited our spine clinic. Sagittal cervical spine MRI showed an increased diffusion signal in the C1–C6 spinal cord and C4/C5 cervical vertebral bodies on T2-weighted images, indicating an infection (Fig. 6). He was admitted to our inpatient department. On the 59th day, the patient presented with severe headache, dyspnea, abdominal distention, and uroschesis, and myodynamia of both lower extremities was zero. He was transferred to the intensive care unit. Later that night, he experienced cardiopulmonary arrest and was resuscitated successfully. On the midday of the 60th day, he again had cardiopulmonary arrest, but resuscitation failed.

## 3 Discussion

In 1950, Forestier and Rotes-Querol named senile ankylosing hyperostosis of the spine to reflect their observations of anterolateral perivertebral ligament calcification featured in the patients.^[1]^ In the late 1970 s, Resnick et al^[3–5]^ discovered that the spine was only 1 of the many possible sites involved in ligamentous ossification; therefore, the term diffuse idiopathic skeletal hyperostosis (DISH) was suggested.^[3]^ DISH is the most commonly used term to describe spinal and extra-spinal ligamentous ossification although Forestier–Rotes-Querol disease is still popular.^[6,7]^

DISH occurs in approximately 14% to 18% of the general population, prevalently in slightly obese and middle-aged or elderly individuals of both sexes.^[8]^ The most common sites of involvement in the spine, in a descending order of incidence, are the lower thoracic (T8–T11), cervical, and lumbar spine,^[3]^ whereas the most common extra-spinal sites are the pelvis, heel, elbow, and knee.^[9,10]^ Dysphagia related to DISH affecting the cervical spine has a reported prevalence of 28%.^[5]^

Large anterior cervical osteophytes causing compression of the esophagus and, more rarely, upper respiratory tract are regularly reported in ENT and pulmonology journals as well as by anesthesiologists as a cause of intubation difficulties.^[7]^ In a patient with dysphagia, a thorough history and careful regional physical examination are essential.^[11]^ The diagnosis of DISH is radiologically confirmed. Conventional plain films of the cervical spine typically show flowing calcification and ossification along the anterior surface.

According to Resnick,^[12]^ the following 3 radiographic criteria are considered diagnostic for the classic spinal involvement of DISH:

(1) presence of flowing hyperostoses located at the anterolateral aspect of at least 4 contiguous vertebral bodies;
(2) relative preservation of the involved intervertebral disk spaces with the absence of significant radiographic changes of degenerative disk disease; and
(3) absence of apophyseal joint ankylosis and sacroiliac joint erosions, sclerosis, or intra-articular osseous fusion.

CT is another useful imaging modality in the diagnosis of DISH as the size and shape of the osteophytes are shown in the esophagus and other important structures. A supplemental study, such as modified barium swallow,^[11,13]^ is an effective tool to evaluate morphologic and functional abnormalities characterizing swallowing, to rule out complications such as aspiration and permit a dynamic introduction and critical assessment of conservative treatment strategies. Initial evaluation with endoscopy procedures remains controversial for the possibility of esophageal wall damage or perforation.^[2,14]^ Wright^[15]^ encountered a case of upper esophageal perforation with endoscopy secondary to cervical osteophytes in which the patient consequently died.

Dysphagia caused by DISH may be due to several factors: direct mechanical compression of the esophagus by large anterior osteophytes; smaller osteophytes located at the sites of esophageal fixation, such as at the level of the cricoid cartilage; inflammation of the periesophageal soft tissue in contact with overlying osteophytes; or esophageal spasm caused by painful osteophytes.^[16]^ This patient's dysphagia caused by DISH was due to inflammation of the periesophageal soft tissue in contact with overlying osteophytes, depending on a 20-day history of dysphagia and hyperemic mucosal tissue of the first laryngoscopy showing.

Patients with dysphagia due to DISH should initially undergo conservative treatment. This treatment includes diet modification, nonsteroidal inflammatory medications, corticosteroids, and muscle relaxants.^[17,18]^ If conservative trials are unsuccessful, surgical resection may be performed. Surgery may not be an ultimate lasting solution. Long-term studies have shown that a relative increase in intervertebral mobility resulting from surgery may accelerate reossification in DISH.^[19]^ To reduce this, it may be necessary to immobilize the involved segments or perform osteophytectomy.^[19,20]^

Patients undergoing endoscopy are at risk of perforation, but this procedure may be necessary to exclude other intrinsic causes of dysphagia, such as esophageal strictures, esophagitis, esophageal webs, motility disorders, tumors, and candidiasis.^[2]^ Such risk may be explained based on the anatomic characteristics. The posterior wall of the pharynx is located in front of the cervical vertebral bodies (C1–C6) with loose connective tissue, and it is continuous with the esophageal wall in front of the C6 vertebral body. The laryngopharynx is parallel to the cervical vertebral bodies (C3–C6). The upper boundary of the larynx is the epiglottis, and the lower boundary is the cricoid cartilage. Therefore, there is a shared channel between the laryngopharynx and larynx in front of the C3–C5 vertebral bodies.^[21]^ The posterior wall of the pharynx is raised and compressed to the larynx by osteophytes. The shared channel is occupied by the bulk osteophytes, and the posterior wall of the pharynx becomes thin. As a result, it is difficult to insert the endoscope into the channel, and perforation most likely occurs when endoscopy or biopsy is performed.

Hypopharyngeal or cervical esophageal perforation is most considered for nonoperative management due to the anatomic confinement of the esophagus by surrounding surgical structures. Appropriate patient selection can achieve a 100% survival rate.^[22,23]^ Patients are maintained on intravenous fluids, NPO for 7 days, and broad-spectrum antibiotics for 5 to 7 days. If patients remain clinically stable, contrast esophagography is performed on the 7th day, and the resumption of oral intake under observation is considered depending on the results.^[24]^ However, pharyngeal perforation with DISH may not be the appropriate patient selection for conservative treatment. The perforation of this patient could not heal completely until 56 days after endoscopy. Such healing is difficult because of the compression and interference from large osteophytes. Therefore, operative management may be an appropriate option for these patients.

## 4 Lessons

DISH involving the cervical spine is a complicated cause of dysphagia. Due to the anatomic variation of the pharynx secondary to DISH, patients undergoing endoscopy are at risk of perforation. If other intrinsic causes of dysphagia have to be excluded with the aid of endoscopy, plain films and CT images should be read carefully first. According to CT images, we need to pay attention to identify the size and shape of the osteophytes shown with the esophagus and other important structures. To minimize the risk of perforation, it is necessary to perform endoscopy extremely carefully, especially biopsy. Once perforation occurs, operative treatment may be more appropriate and effective.

## Author contributions

**Supervision:** Xueju Li, Cunping Wang.
